# CRISPR/Cas9-Targeted *Myostatin* Deletion Improves the Myogenic Differentiation Parameters for Muscle-Derived Stem Cells in Mice

**DOI:** 10.3390/jdb13010005

**Published:** 2025-02-11

**Authors:** Mohamed I. Elashry, Victoria C. Schneider, Manuela Heimann, Sabine Wenisch, Stefan Arnhold

**Affiliations:** 1 Institute of Veterinary Anatomy, Histology and Embryology, Justus Liebig University of Giessen, 35392 Giessen, Germany; vikcath@hotmail.com (V.C.S.); manuela.heimann@vetmed.uni-giessen.de (M.H.); stefan.arnhold@vetmed.uni-giessen.de (S.A.); 2 Clinic of Small Animals, Institute of Veterinary Anatomy, Histology and Embryology, Justus Liebig University of Giessen, 35392 Giessen, Germany; sabine.wenisch@vetmed.uni-giessen.de

**Keywords:** myostatin, muscle stem cells, CRISPR/Cas9, myogenic differentiation

## Abstract

Skeletal muscle plays a pivotal role in physical activity, protein storage and energy utilization. Skeletal muscle wasting due to immobilization, aging, muscular dystrophy and cancer cachexia has negative impacts on the quality of life. The deletion of myostatin, a growth and differentiation factor-8 (GDF-8) augments muscle mass through hyperplasia and hypertrophy of muscle fibers. The present study examines the impact of myostatin deletion using CRISPR/Cas9 editing on the myogenic differentiation (MD) of C2C12 muscle stem cells. A total of five myostatin loci were targeted using guided RNAs that had been previously cloned into a vector. The clones were transfected in C2C12 cells via electroporation. The cell viability and MD of myostatin-edited clones (Mstn^−/−^) were compared with C2C12 (Mstn^+/+^) using a series of assays, including MTT, sulforhodamine B, immunocytochemistry, morphometric analysis and RT-qPCR. The clones sequenced showed evidence of nucleotides deletion in Mstn^−/−^ cells. Mstn^−/−^ cells demonstrated a normal physiological performance and lack of cytotoxicity. Myostatin depletion promoted the myogenic commitment as evidenced by upregulated MyoD and myogenin expression. The number of MyoD-positive cells was increased in the differentiated Mstn^−/−^ clones. The Mstn^−/−^ editing upregulates both mTOR and MyH expression, as well as increasing the size of myotubes. The differentiation of Mstn^−/−^ cells upregulates ActRIIb; in contrast, it downregulates decorin expression. The data provide evidence of successful CRISPR/Cas9-mediated myostatin deletion. In addition, targeting myostatin could be a beneficial therapeutic strategy to promote MD and to restore muscle loss. In conclusion, the data suggest that myostatin editing using CRISPR/Cas9 could be a potential therapeutic manipulation to improve the regenerative capacity of muscle stem cells before in vivo application.

## 1. Introduction

Skeletal muscle plays a pivotal role in regulating several fundamental physiological processes, including body locomotion, energy storage and metabolism. Muscle wasting occurs with aging (sarcopenia) [1], imbalanced caloric intake, immobilization, neurodegenerative diseases, such as spinal muscular atrophy (SMA) [2,3], and muscular dystrophy that leads to muscle mass loss, physical disability and poor quality of life. Duchenne muscular dystrophy (DMD) is a severe muscle disorder caused by absence of dystrophin, a protein that anchors the contractile elements of the muscle fiber to the extracellular matrix, which results in progressive muscle degeneration [4,5].

Skeletal muscle-derived stem cells (MSCs), also known as satellite cells and first described as mononuclear cells, lie between the basal lamina and the plasmalemma of muscle fibers [6]. These cells are capable of self-renewal and differentiation into various mesenchymal lineages, including muscle, bone and fat [7]. Following muscle injury, a chemotactic response of growth factors and cytokines, including fibroblast growth factors (FGFs), insulin-like growth factor (IGFs), transforming growth factor-beta (TGF-β), hepatocyte growth factor (HGF), tumor necrosis growth factor-alpha (TNF-α) and interleukin-6 (IL-6), results in the recruitment and subsequent activation of MSCs [8]. The activated myoblasts express the proliferation markers myoblast determination protein 1 (MyoD) and myogenic regulatory factor 5 (Myf5); however, the differentiating cells express myogenic regulatory factor 4 (Mrf4) and myogenin [9,10,11]. Differentiating cells fuse to form multinucleated syncytium, referred to as myotubes [12].

Myostatin (Mstn), also known as growth and differentiation factor-8 (GDF-8), is a member of the TGF-β superfamily and is the main regulator of skeletal muscle development [13]. Mstn signaling has been demonstrated to downregulate the transcription of muscle genes, including Pax3, MyoD and Myf5, to reduce cell proliferation and to promote cell differentiation [14]. Mstn is synthesized in an inactive precursor, which requires a proteolytic cleavage to release the active domain [15,16]. The active Mstn ligand binds to activin receptor type IIb (ActRIIb), thereby triggering an intracellular signaling cascade, including the phosphorylation of Smad2 and Smad3 transcription factors and the nuclear translocation of Smad4, which modulates the transcription of the myogenic genes via upregulation of p21 expression to inhibit cell proliferation [17].

The inhibition of Mstn represents a promising approach to enhancing muscle mass and offers a potential therapeutic avenue for muscle wasting. A substantial body of evidence has demonstrated that the absence of Mstn or the blocking of its activity increases skeletal muscle mass, as observed in canine [18], sheep [19], cattle [20], horse [21], pig [22] and human studies [23]. Several methods having the potential to inhibit Mstn have been developed. Briefly, follistatin has been shown to antagonize and block Mstn activity [24,25]. The inhibition of metalloproteases has the potential to interfere with the Mstn signaling pathway by preventing the cleavage of the propeptide from the active Mstn protein [26]. The overexpression of the propeptide has been demonstrated to exert an inhibitory effect on Mstn activity, specifically through the inhibition of the binding of the active Mstn to actRIIb [27]. Using oligonucleotides to induce exon skipping of the Mstn RNA resulted in a production of truncated or improper mRNA expression [28,29]. A study demonstrated that the combined inhibition of ActRIIb and exon skipping for dystrophin using an adeno-associated vector enhanced the tetanic and specific forces in the mdx mouse model for Duchenne muscular dystrophy, indicating a potential therapeutic strategy for muscle wasting diseases [30]. Furthermore, the cross-breeding of Mstn knockdown with mdx mice demonstrated an enhanced muscle regeneration, characterized by augmented muscle mass and diminished fibrosis in the diaphragm [31].

The recent emergence of CRISPR/Cas9 (clustered regularly interspaced short palindromic repeats CRISPR-associated proteins) gene editing has led to the development of novel technology for the overexpression and deletion of specific genes. CRISPR/Cas9 are adaptive immune systems that are used by most Archaea to provide a resistant mechanism against invading elements such as viruses and plasmids [32,33,34]. The disruption of Mstn using CRISPR/Cas9 has been successfully demonstrated in goats [35], sheep [36], pigs [37,38], rabbits [39] and chicks [40]. The objective of the present study is to investigate the impact of Mstn inhibition through CRISPR/Cas9 gene editing on the myogenic differentiation (MD) capacity of the C2C12 mouse myoblast cell line. A total of five loci of myostatin were subjected to editing using designed guide RNA primers. The target loci were cloned into CRISPR/Cas9-equipped vectors, then introduced into the C2C12 mouse myoblast cell line using electroporation. The results demonstrate the successful editing of the Mstn gene, as evidenced by the presence of nucleotides deleted, as well as absence of Mstn precursor in Mstn^−/−^ cells. The data analysis demonstrates that there is no alteration in the cell viability of the edited clones. Mstn^−/−^ precursors demonstrated enhanced myogenic commitment, elevated protein synthesis and expression of contractile protein, which collectively are indicative of promoted MD. The Mstn-edited clones exhibited augmented myotube size, suggesting a hypertrophy of the muscle fiber as shown in the knockout mice. The data provide evidence of altered ActRIIb, mTOR and decorin expression in the absence of myostatin, which suggests the existence of compensatory gene regulation. This data demonstrate the possibility of myostatin editing using CRISPR/Cas9, which could be a potential therapeutic application for both editing and enhancing the regenerative capacity of MSCs prior to in vivo application. Furthermore, the inhibition of Mstn represents a promising avenue for the development of novel therapeutic strategies for both muscle atrophy and muscle regeneration.

## 2. Materials and Methods

### 2.1. Cell Culture

The study was conducted by Justus Liebig University of Giessen institutional ethics committee Nr. V 54–19 c 20 15 h 02 GI 18/1 kTV 1/2018 and the Regierungspräsidium Giessen (approval code: GI 20/10 Nr. 105/2014), Date of approval: 16 March 2018). The C2C12 cell line, skeletal muscle-derived myoblasts, is an immortalized satellite cell line isolated from mice. C2C12 cells were cultivated in a growth medium composed of 4.5 g/L of Dulbecco’s Modified Eagle’s Medium (DMEM, Gibco, Life Technologies, Darmstadt, Germany), supplemented with 10% fetal calf serum (FCS, Capricorn, Ebsdorfergrund, Germany) and 1% penicillin/streptomycin (P/S, Gibco, Life Technologies, Darmstadt, Germany) under standard culture conditions at 37 °C and 5% CO_2_. The medium was replenished every two to three days. Upon reaching 60% confluency, the cells were washed in prewarmed phosphate-buffered saline (PBS, Gibco, Life 138 Technologies, Darmstadt, Germany) for five min, then detached after incubation with TrypLE^TM^ (Gibco, Life Technologies, Darmstadt, Germany) for five min at 37 °C. The enzymatic reaction was terminated by adding an equal volume of fresh medium. The cells were centrifuged at 240× *g* for three min, then the supernatant was discarded, and the cell pellet was suspended in fresh medium. The cells were subsequently counted using a Neubauer counting chamber, then subcultivated based on the experimental setup. Only cells from passages 13–15 were used for all experiments. The cells in the growth medium were either subcultivated for the next experiment or cryopreserved as 1 × 10^6^ cells per mL in freezing medium under −196 °C in liquid nitrogen.

### 2.2. CRISPR/Cas9 Mstn Editing

The target deletion site could be created using various target design software, including RGEN Cas-Designer (Free tool software 2.X http://www.rgenome.net/cas-designer/portable, accessed on 6 February 2025), CRISPRdirect [41] (free editing software, http://crispr.dbcls.jp, accessed on 6 February 2025), GT-Scan (https://bioinformatics.csiro.au/gt-scan/, accessed on 6 February 2025) and CRISPR.mit.edu (https://www.zlab.bio/resources, accessed on 6 February 2025). The experimental setup and the sequence of the targeted Mstn loci were simplified (Supplementary Figure S1a,b). Five gRNAs were designed for each Mstn locus, and the sequence of the used oligonucleotides is listed in Table 1. The guide RNA was transferred to the pX459 vector containing the human U6 promoter, the nuclease hSpCas9 (Streptococcus pyogenes Cas9 optimized for human codon) and a puromycin resistance gene. A hybrid oligoduplex comprising the designed oligonucleotides and the target sequences was prepared. To prevent re-ligation, a calf intestinal phosphatase (CIP) was added. The DNA purification was conducted using the PeqLab DNA purification column according to the manufacturer’s instructions. The purified DNA was eluted in ultrapure H_2_O. In parallel, a similar setup without target DNA inserts in the vector referred to as pX459 served as the negative control. After ligation, the plasmid was transfected into the competent cells. The probes were plated on an LB plate supplemented with 100 µg/mL ampicillin (Carl Roth, Karlsruhe, Germany), then incubated at 37 °C overnight. A test PCR was conducted on the bacterial colonies to confirm the incorporation of the appropriate guide DNA into the vector. The results were evaluated through 2% agarose gel electrophoresis (Bioline, Luckenwalde, Germany) at 180 V for 20 min.

Plasmid purification was performed using a basic DNA purification kit (Roboklon, Berlin, Germany) according to the manufacturer’s instructions. The DNA was eluted in 25–50 µL of ultrapure H_2_O. Purification of a large volume of plasmid, a midi plasmid preparation, was carried out using the endotoxin-free plasmid DNA purification kit NucleoBond^®^ (Th. Geyer, Renningen, Germany) according to the manufacturer’s instructions. The transfection was conducted using electroporation (Neon^TM^ Transfection System, Thermo Fischer Scientific, Waltham, MA, USA). Briefly, 5 × 10^5^ C2C12 cells and 5 µg of DNA were suspended in 100 µL of Buffer R, then loaded in a Neon tip. The Neon tip was transferred to a Neon tube loaded with 3 mL of E2 buffer. Then, the tube was exposed to a high-voltage pulse of 1650 V for 10 ms in the Neon Transfection System. The cells were incubated in a cultivation medium without P/S in 10 cm diameter cell culture plates at 37 °C. Forty-eight hours after electroporation, the cells were detached, then cultivated in 1:5 and 1:10 dilutions with growth medium supplemented with 2 µg/mL puromycin (Enzo Life Sciences GmbH, Lörrach, Germany) for 10 days. The remaining cells were frozen in liquid nitrogen. Single-cell clones were isolated via the filter disc cell detachment technique. A minimum of ten single-cell clones per target site were isolated, then processed for either expansion or freezing. The genomic DNA of the individual cells was isolated using a Tissue DNA Purification Kit (Roboklon Berlin, Germany) according to the manufacturer’s instructions, sequenced through a third-party sequencer (Microsynth Seq lab GmbH, Göttingen, Germany) and analyzed with the software Serial Cloner 2.6.1 and Snap Gene Viewer 8. The sequence analysis was conducted to ascertain whether mutations were in the sequence of the Mstn compared to the original sequence provided by NCBI (reference sequence: NM_010834.3).

### 2.3. Cell Viability Assay

The (3-(4,5-dimethylthiazol-2-yl)-2,5-diphenyltetrazolium bromide MTT assay (Carl Roth, Karlsruhe, Germany) was employed to determine the metabolic activity of the cells. The cells of Mstn^+/+^ and Mstn^−/−^-edited clones were cultivated at a density of 2 × 10^4^ in 24-well plates (VWR, Darmstadt, Germany) for 24 and 48 h in a growth medium. The medium was removed, and the cells were incubated with MTT solution diluted 1:10 in the cultivation medium at 37 °C and 5% CO_2_ for 2 h. The yellow color of the MTT reagent was converted into a blue-purple formazan product. The MTT solution mixture was then removed, and the cells were incubated with 200 μL per well of dimethylsulfoxide for 10 min at RT on a shaker. A volume of 100 µL from each experimental condition was transferred into a 96-well plate in triplicate. The formation of formazan was quantified by measuring the absorbance at 570 nm using a microplate reader equipped with Magellan TM Data Analysis V2.30 Software (Tecan, Männedorf, Switzerland).

### 2.4. Sulforhodamine B Assay (SRB)

The SRB assay was reported to semi-quantify the total protein content, which is indicative of cell growth [42]. The cells from all experimental groups were cultivated at a density of 2 × 10^4^ in a 24-well plate in a growth medium for 24 and 48 h. Subsequently, the cells were fixed in 4% paraformaldehyde (PFA, Carl Roth, Karlsruhe, Germany) for 10 min, then washed twice with distilled water. Afterwards, the cells were incubated with 300 µL of 0.4% SRB sodium salt (Th. Geyer, Renningen, Germany) diluted with 1% acetic acid (Merck, Darmstadt, Germany) for 10 min at RT. Following a washing step with 1% acetic acid until a clear supernatant was observed, the cells were incubated with 250 µL per well of unbuffered TRIS base on a shaker for 10 min. For each experimental condition, a volume of 100 μL was pipetted in triplicate into a 96-well plate. The SRB intensity was determined at 565 nm absorbance, as described for the MTT assay using the microplate reader (Tecan, Männedorf, Switzerland).

### 2.5. Myogenic Differentiation

For MD induction, cells were seeded at a density of 2 × 10^4^ in a 24-well plate. Upon reaching 80% confluency, the cells were incubated with a differentiation medium, comprising DMEM-LG, 2% horse serum (HS, Millipore, Darmstadt, Germany) and 1% P/S for up to seven days. Regular medium changes were conducted every 2–3 days. The plates were divided into two groups, C2C12 without genetic manipulation (Mstn^+/+^) and CRISPR/CAS9 Mstn-edited clones (Mstn^−/−^), including pMH17-7, pMH17-9, pMH18-19, pMH18-20, pMH19-21, pMH19-27, pMH20-36, pMH20-38, pMH21-48 and pMH21-50. Cells transfected with a pX459 vector lacking DNA inserts were cultivated in parallel to serve as the negative control. Following the requisite cultivation period, the cells were fixed in 4% PFA for 10 min and stored in PBS at 4 °C until further experiments.

### 2.6. RT-qPCR

Following seven days of MD, cells from both Mstn^+/+^ and Mstn^−/−^-edited clones were lysed for the purpose of RNA isolation, which was conducted using the GenElute Mammalian RNA Miniprep Kit (Sigma-Aldrich, Taufkirchen, Germany) in accordance with the instructions provided by the manufacture. The harvested RNA was quantified through photometric analysis using a Nanodrop spectrophotometer. Approximately 810 ng of RNA per experimental condition was digested in 1.2 units of recombinant DNase I (Sigma-Aldrich, Taufkirchen, Germany) at 37 °C for 30 min. Following the DNAse I digestion, cDNA synthesis was conducted using MultiScribe^TM^ Reverse Transcriptase (Thermo Fisher Scientific, Dreieich, Germany). The resulting cDNA was employed for subsequent qualitative or quantitative gene expression analysis. For qualitative PCR, a volume of 2.5 μL of cDNA was pipetted into a pre-prepared PCR mix, and a protocol of thermal cycling was carried out. This involved an initial denaturation step at 95 °C for 5 min, followed by 30 s at 94 °C, 30 s at 60 °C, 30 s at 72 °C and a final extension at 72 °C for 1 min. For RT-qPCR, one μL of cDNA from three independent experimental setups (*n* = 3) was pipetted in triplicate in PCR microplates containing the primer of interest and the GoTaq^®^ qPCR Master Mix (Promega, Walldorf, Germany) in a CFX96 Touch Real-Time PCR Detection System (Biorad, Feldkirchen, Germany). The relative expression of myosin heavy chain 1 (MyH1), myosin heavy chain 2 (MyH2), myosin heavy chain 7 (MyH7), decorin, myostatin, activin receptor type IIb (ActRIIb), myogenin, MyoD and mammalian target of Rapamycin (mTOR) in proliferated and differentiated Mstn^+/+^ and Mstn^−/−^-edited clones was quantified. The RT-qPCR data were analyzed using the 2^−ΔΔCt^ method as described by [43]. The primers used are listed in Table 2. An analysis of a single clone of either pX459- or pX459-transfected C2C12 cells was conducted in parallel in proliferated and differentiated states as negative control. The *18S* served as an endogenous reference gene.

### 2.7. Phalloidin Staining

Phalloidin is a commonly used staining agent for the cytoskeleton’s actin filaments. The myoblasts of both experimental groups were cultivated at a density of 2 × 10^4^ cells per well on a glass coverslip with the MD medium for seven days. Following three consecutive washes in PBS for 5 min each, the cells were incubated with a 1:40 dilution of phalloidin staining (Sigma-Aldrich, Steinheim, Germany) in PBS for 30 min in the dark. Similarly, the cells were washed three times for 5 min in PBS, after which the nuclei were visualized with 4′,6-diamidine-2-phenylindole (DAPI, Carl Roth, Karlsruhe, Germany) diluted 1:5000 in PBS for 5 min. The cells were washed twice for 5 min with PBS, then mounted with DABCO-Mowiol (Carl Roth, Karlsruhe, Germany). They were subsequently examined and photographed under a fluorescence microscope equipped with the “AxioCam MRm Rev.3” camera and the “AxioVision Image Analysis 4.8.2” software.

### 2.8. Immunocytochemistry

The cells were seeded at a density of 2 × 10^4^ cells per well in 24-well plates. The plates were divided into two experimental groups cultivated in either growth (proliferation, PF) medium up to 48 h or in differentiation (DF) medium with 2% horse serum up to seven days. The cells were fixed in 4% PFA for 10 min. Subsequently, the cells were washed three times for 5 min in PBS. They were then permeabilized with 0.1% Tween 20 in PBS for 10 min. Following three consecutive washes in PBS for 5 min, the non-specific bindings were blocked by incubating the cells with 10% goat serum for 30 min. The cells were incubated with primary antibodies, anti-mouse MyoD (1:100, BD Bioscience, Heidelberg, Germany), anti-mouse myogenin (1:100, Santa Cruz, Biotechnology, Heidelberg, Germany) and anti-mouse MyH F-59 (1:200, Santa Cruz, Biotechnology, Heidelberg, Germany), diluted in blocking buffer at 4 °C overnight. The cells were washed three times for 5 min each with washing buffer. Subsequently, the primary antibodies were visualized by adding Cy3-goat anti-mouse IgG secondary antibody (diluted 1:200, Dianova, Hamburg, Germany) in the dark at RT for one h. After three further washes for 5 min, the nuclei were stained in DAPI for 5 min. Following three washes for 10 min each, the cells were mounted with DABCO-Mowiol, then examined and photographed under a fluorescence microscope equipped with an “AxioCam MRm Rev.3” camera and the “AxioVision Image Analysis 4.8.2” software. To analyze the immunocytochemistry staining, eight random images at 32× magnification per experimental group were selected and analyzed using ImageJ 1.52e. The number of MyoD- and myogenin-positive cells in Mstn^+/+^ and Mstn^−/−^-edited clones was quantified. Morphometric parameters, including the thickness of the myotubes and fusion index by dividing the number of nuclei in myotubes by the total number of nuclei in the microscopic field, were assessed.

### 2.9. Statistical Analysis

The data output of MTT, sulforhodamine B, immunocytochemistry, morphometric analysis and RT-qPCR for cells from Mstn^+/+^ and Mstn^−/−^-edited clones following seven days of MD were subjected to statistical analysis. The data were collected from three independent experimental setups (*n* = 3). The MTT assay, SRB assay and relative gene expression data were evaluated using a two-way ANOVA. The Tukey test was considered a post-hoc test. The counting of MyoD, myogenin positive cells during the proliferation (PF) and differentiation (DF) phases for Mstn^+/+^ and Mstn^−/−^-edited clones was conducted using a two-way ANOVA followed by Tukey’s test as a post-hoc test. The statistical analysis was conducted using the Snap Gene Viewer 4.2.11 software and GraphPad Prism Version 9.2.0 software. For the remaining analysis, including the measurement of the thickness of the myotubes and the differentiation index, an unpaired *t*-test was carried out. The data analysis was presented as mean ± SEM, and *p* values ≤ 0.05 were considered statistically significant.

## 3. Results

### 3.1. Myostatin Loci Targeting and Editing Using CRISPR/Cas9

In order to generate Mstn^−/−^ clones and thereby validate the importance of myostatin (Mstn) on muscle stem cell regeneration, the CRISPR/Cas9 method was employed. This novel technology represents one of the most accurate and straightforward gene editing methods currently available. The vectors cloned with DNA inserts are referred to as pMH17, pMH18, pMH19, pMH20 and pMH21. Clone sequencing revealed successful nucleotide deletions in the Mstn^−/−^-edited clones; the data analysis of the clones revealed a large deletion of 243 nucleotides in Mstn^−/−^ 17–9, 51 nucleotides in Mstn^−/−^ 17–7, 38 nucleotides in Mstn^−/−^ 21–48 and 23 nucleotides in Mstn^−/−^ 18–19. However, a small deletion of a single nucleotide in Mstn^−/−^ 18–20, three nucleotides in Mstn^−/−^ 19–21 and five nucleotides in Mstn^−/−^ 20–38 were detected (Table 3 and Supplementary Figure S2).

### 3.2. Assessment of Cell Viability in the Mstn^−/−^ Cells

**Figure 1:**
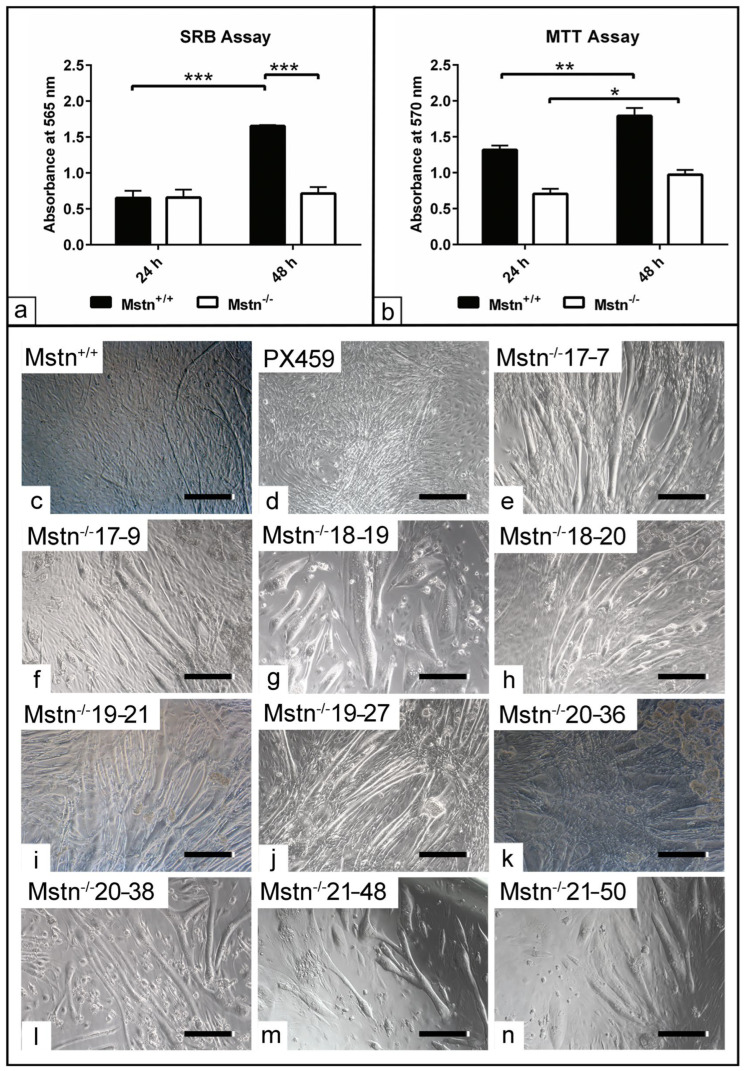
Assessment of cell viability and myogenic differentiation capacity of the myostatin-edited clones (Mstn^−/−^) compared to control C2C12 (Mstn^+/+^). (**a**) Semi-quantitative measurement of total protein content using SRB assay (*n* = 3) demonstrates the cell numbers following 24 h and 48 h in growth medium. The cells were fixed with 4% paraformaldehyde for 10 min and then stained with 0.4% sulforhodamine B (SRB) solution in 1% acetic acid for 10 min. The absorbance was measured at 565 nm. (**b**) Semi-quantitative measurement of cell viability using MTT assay (*n* = 3) shows the metabolic activity for 24 h and 48 h in growth medium. The absorbance was measured at 570 nm. (**c**–**k**) Representative phase contrast images display myotube formation indicative for myogenic differentiation after seven days for Mstn^−/−^ cells and Mstn^+/+^ cells. (**c**) Mstn^+/+^ control. (**d**) pX459 transfected cells without target DNA insert. (**e**) Mstn^−/−^ 17–7 edited clone. (**f**) Mstn^−/−^ 17–9 edited clone. (**g**) Mstn^−/−^ 18–19 edited clone. (**h**) Mstn^−/−^ 18–20 edited clone. (**i**) Mstn^−/−^ 19–21 edited clone. (**j**) Mstn^−/−^ 19–27 edited clone. (**k**) Mstn^−/−^ 20–36 edited clone. (**l**) Mstn^−/−^ 20–38 edited clone. (**m**) Mstn^−/−^ 21–48 edited clone. (**n**) Mstn^−/−^ 21–50 edited clone. All data are presented as the mean ± SEM. * *p* < 0.05, ** *p* < 0.01, *** *p* < 0.001. Scale bar = 100 µM.

To examine the Mstn^−/−^ cells of the edited clones compared to Mstn^+/+^ cells, a semi-quantitative analysis of the cell number and viability was conducted up to 48 h. The data analysis showed no alteration in total protein content between the Mstn^−/−^ cells and Mstn^+/+^ after 24 h in the growth medium. Although the Mstn^+/+^ cells demonstrated an increased cell number at 48 h (*p* < 0.001), the Mstn^−/−^ cells exhibited no statistically significant differences compared to the previous time point. At 48 h, the Mstn^+/+^ cells showed a higher cell number (*p* < 0.001) compared to the Mstn^−/−^ cells. The data analysis demonstrated a significant interaction (*p* < 0.001) between the effect of mutation at different time points (Figure 1a). By evaluating the cell viability, the data analysis revealed that the metabolic activity of the cells was enhanced at 48 h (*p* < 0.01 and *p* < 0.05), in comparison to 24 h for both Mstn^+/+^ and Mstn^−/−^ cells (Figure 1b). Subsequently, the cells of various clones were induced to differentiate for up to seven days to identify the most suitable clones for gene expression analysis based on their morphological observation. The data demonstrated an increase in the myotube formation indicative of MD in the Mstn^−/−^-edited clones, including Mstn^−/−^ 17–7, Mstn^−/−^ 17–9, Mstn^−/−^ 18–19, Mstn^−/−^ 18–20, Mstn^−/−^ 19–21, Mstn^−/−^ 19–27, Mstn^−/−^ 20–36, Mstn^−/−^ 20–38, Mstn^−/−^ 21–48 and Mstn^−/−^ 21–50 compared to those cells of either only Mstn^+/+^ or Mstn^+/+^ transfected with naked pX459 vector without DNA insert (Figure 1c–n). To examine whether CRISPR/Cas9 editing alters the levels of Mstn protein, a western blot analysis of cell lysates after seven days in differentiation medium was carried out. The results demonstrated the absence of Mstn precursor band at 52 KD for the Mstn-edited clones; including Mstn^−/−^ 18–19, Mstn^−/−^ 20–38 and Mstn^−/−^ 21–48 compared to either Mstn^+/+^ cells or Mstn^+/+^/pX459 transfected cells without DNA insert. In addition, cell lysates of Mstn^−/−^ 18–15 edited clones showed no alteration compared to control groups (Supplementary Figure S3).

### 3.3. Assessment of the Myogenic Relative Gene Expression in the Mstn^−/−^ Cells

**Figure 2:**
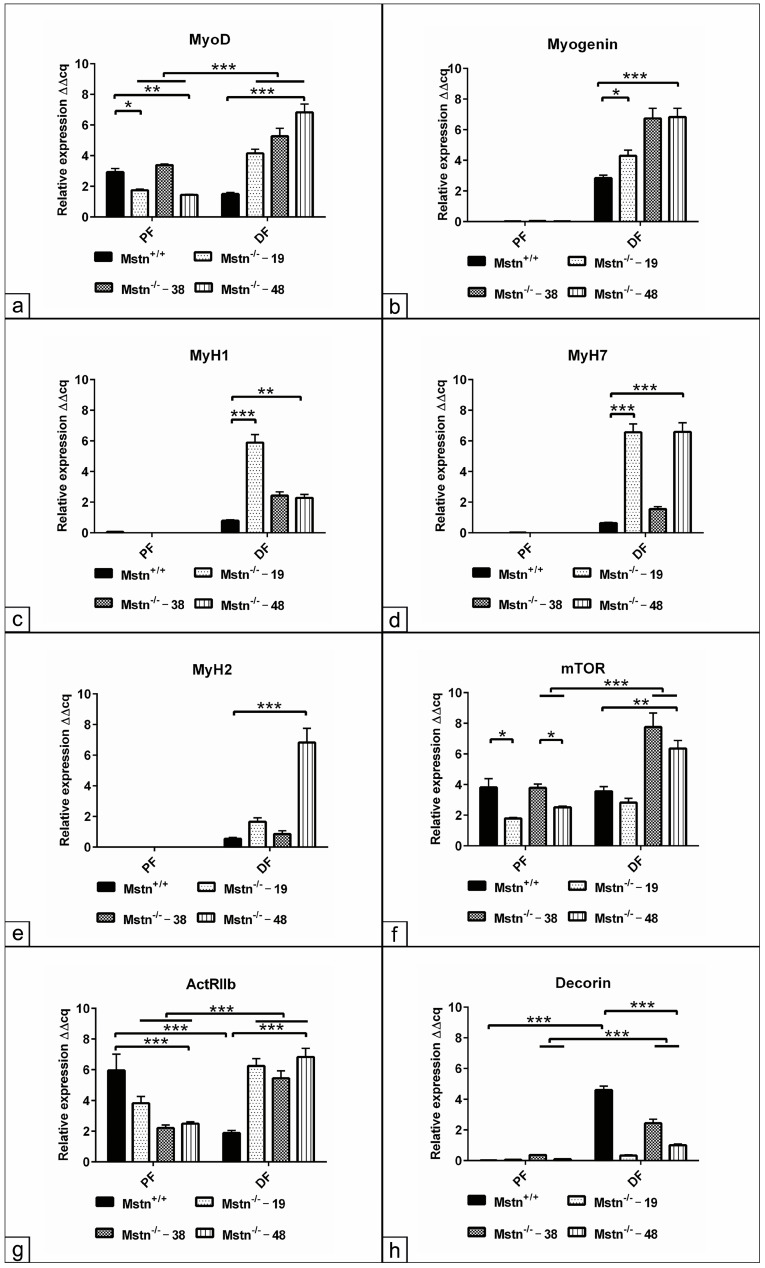
(**a**–**h**) Assessment of the myogenic relative gene expression in the Mstn^−/−^ cells. Cells of three Mstn^−/−^-edited clones, including Mstn^−/−^ 19, Mstn^−/−^ 38 and Mstn^−/−^ 48, in comparison to Mstn^+/+^ control, were cultivated in myogenic differentiation conditions for seven days. Quantitative RT-PCR was conducted using cell lysates from all experimental groups. The relative expression of (**a**) MyoD, (**b**) myogenin, (**c**) myosin heavy chain 1 (MyH1), (**d**) MyH7, (**e**) MyH2, (**f**) mammalian target of Rapamycin (mTOR), (**g**) activin receptor IIb (ActRIIb) and (**h**) decorin are presented. The data were analyzed using the 2^−ΔΔCt^ method. All data are presented as the mean ± SEM. * *p* < 0.05, ** *p* < 0.01, *** *p* < 0.001.

To examine whether Mstn CRISPR/Cas9 editing alters the myogenic relative markers expression, the quantification of MyoD, myogenin and MyH for three selected clones, including Mstn^−/−^ 18–19, Mstn^−/−^ 20–38 and Mstn^−/−^ 21–48, were analyzed. In the growth medium (proliferation, PF), the cells of the Mstn^−/−^ 18–19 and Mstn^−/−^ 21–48 clones exhibited a downregulation of *MyoD* expression (*p* < 0.05 and *p* < 0.01), while no change was observed in the Mstn^−/−^ 20–38 cells compared to the Mstn^+/+^ cells. Following seven days in differentiation (DF) medium, the cells of all Mstn^−/−^ clones exhibited sustained upregulated *MyoD* expression, not only in comparison to Mstn^+/+^ cells (*p* < 0.001) at the same time point but also in comparison to Mstn^−/−^-edited clones (*p* < 0.001) in PF medium (Figure 2a). The expression of the differentiation marker myogenin demonstrated a moderate upregulation in the Mstn^−/−^ 18–19 clone (*p* < 0.05) and a more pronounced two-folds increases in the Mstn^−/−^ 20–38 and Mstn^−/−^ 21–48 clones (*p* < 0.001) compared to Mstn^+/+^ cells (Figure 2b). The quantification of the contractile protein revealed an increase in *MyH1* (intermediate fast contraction) expression in Mstn^−/−^ 18–19 (*p* < 0.001), Mstn^−/−^ 20–38 and Mstn^−/−^ 21–48 (*p* < 0.01) compared to Mstn^+/+^ cells after seven days. Meanwhile, *MyH7* (slow contraction) was upregulated in Mstn^−/−^ 18–19 and Mstn^−/−^ 21–48 (*p* < 0.001) cells compared to Mstn^+/+^ counterparts (Figure 2c,d). Additionally, the expression of MyH2 (intermediate fast contraction) was only detected in Mstn^−/−^ 21–48 cells (Figure 2e). Subsequently, the expression of mTOR was quantified to assess whether Mstn deletion modulates protein synthesis. The data analysis revealed a reduction of mTOR expression in Mstn^−/−^ 18–19 and Mstn^−/−^ 21–48 clones (*p* < 0.05) under PF conditions. However, after seven days, a doubling of *mTOR* expression was observed in Mstn^−/−^ 20–38 and Mstn^−/−^ 21–48 clones (*p* < 0.01) either compared to the Mstn^+/+^ cells or the same clone under PF conditions (*p* < 0.001, Figure 2f). To ascertain whether Mstn editing affects the expression of its own receptor, the analysis revealed a reduction in ActRIIb expression across all Mstn^−/−^-edited clones in PF medium (*p* < 0.001). In contrast, in DF conditions, a two-fold upregulation was markedly detected in all Mstn^−/−^-edited clones (*p* < 0.001) compared to Mstn^+/+^ (Figure 2g). Decorin plays a pivotal role in modulating the active Mstn ligand within the extracellular matrix; thus, we examined whether Mstn editing alters decorin expression. The analysis demonstrated an upregulated decorin expression in the differentiated Mstn^+/+^ cells (*p* < 0.001) compared to those cells in the PF condition. Conversely, a reduction in decorin expression was observed in all differentiated Mstn^−/−^-edited clones (*p* < 0.001) compared to the corresponding condition in Mstn^+/+^ cells (Figure 2h).

### 3.4. Evaluation of the Myogenic Differentiation Using Immunocytochemistry

**Figure 3:**
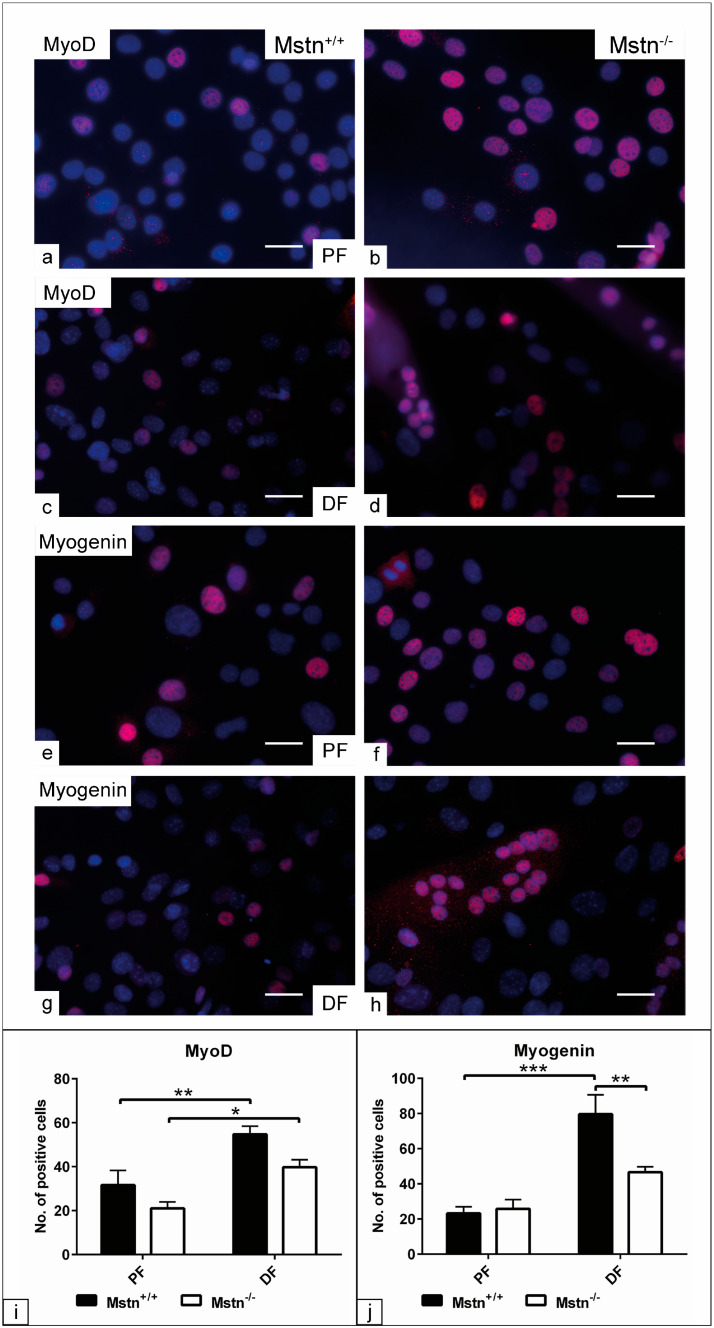
Evaluation of myogenic differentiation using immunocytochemistry. The cells of the Mstn^+/+^ control and the Mstn^−/−^-edited clone were fixed in 4% paraformaldehyde for 10 min following cultivation in either growth medium (proliferation, PF) or differentiation medium (DF) for up to seven days. (**a**–**h**) Immunofluorescence images demonstrate MyoD-positive cells (red, **a**–**d**) and myogenin-positive cells (**e**–**h**) under PF and DF conditions. Cells processed without adding primary antibodies served as a negative control. Nuclei were visualized using DAPI (blue). (**i**) Average number of MyoD-positive cells in random microscopic field (*n* = 8) for Mstn^+/+^ control and Mstn^−/−^-edited clone. (**j**) Average number of myogenin-positive cells (*n* = 8) for Mstn^+/+^ control and Mstn^−/−^-edited clone. All data are presented as the mean ± SEM. * *p* < 0.05, ** *p* < 0.01, *** *p* < 0. 001. Scale bar = 20 µm.

The effect of Mstn editing on the myogenic relative markers indicative for MD potential was examined using immunocytochemistry (Figure 3a–h). The number of MyoD-positive cells in the differentiated cells was increased in both Mstn^+/+^ and Mstn^−/−^ cells (*p* < 0.01 and *p* < 0.05), in comparison to those cells in the proliferation (PF) condition. No significant alteration in the MyoD-positive cells was identified when comparing both genotypes (Figure 3i). The counting of myogenin-positive cells demonstrated no detectable difference between the two groups under PF conditions. In contrast, the differentiated cells exhibited an increase in the number of myogenin-positive cells in the Mstn^+/+^ group, either compared to the growth condition (*p* < 0.001) or compared to the Mstn^−/−^ cells (*p* < 0.01). The counting of myogenin-positive cells in the Mstn^−/−^ group demonstrated no alteration when comparing the growth with differentiation conditions (Figure 3j).

### 3.5. Morphometric Analysis

**Figure 4:**
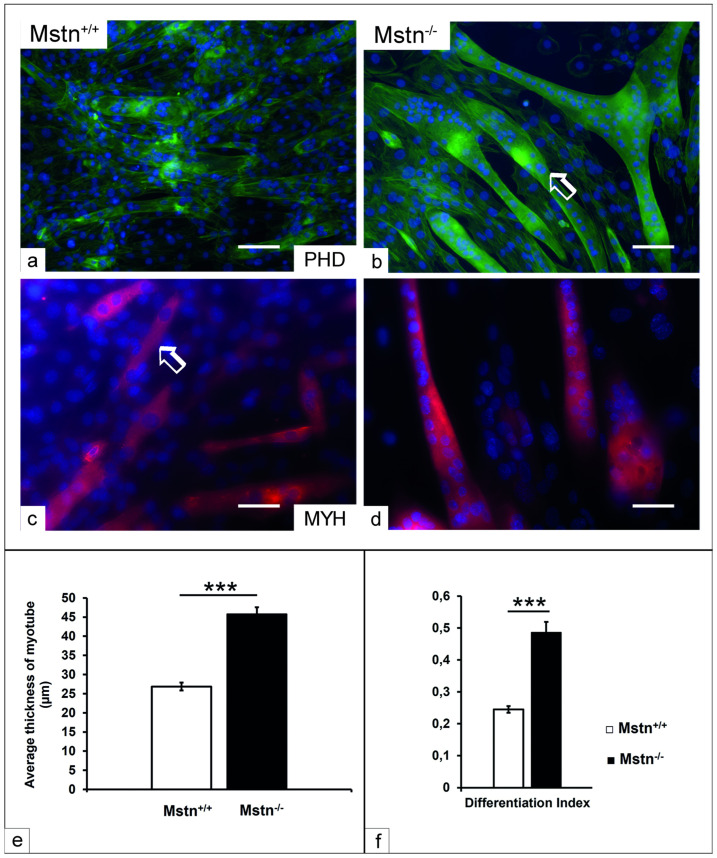
Assessment of myogenic differentiation using morphometric analysis. The cells of the C2C12 control (Mstn^+/+^) and the myostatin-edited clone (Mstn^−/−^) were differentiated for up to seven days with a myogenic differentiation medium. (**a**,**b**) Representative image of myotube formation for Mstn+/+ and Mstn^−/−^ differentiated cells stained in green with phalloidin (PHD, arrow). (**c,d**) Immunocytochemistry of myosin heavy chain (MYH) demonstrates the morphology of myotube (arrow, red) for Mstn^+/+^ and Mstn^−/−^ differentiated cells. (**e**) Morphometric analysis of the thickness of the myotube for Mstn^−/−^ 18–19, Mstn^−/−^ 20–38 and Mstn^−/−^ 21–48 Mstn-edited clones (*n* = 8) compared to Mstn^+/+^. (**f**) Cell differentiation/fusion index refers to the capacity of cell fusion, which is calculated by dividing the number of nuclei per myotube by the total number of nuclei in the same microscopic field. Cells processed without adding primary antibodies served as negative control. Nuclei were counterstained with DAPI (blue). All data are presented as the mean ± SEM. *** *p* < 0. 001. Scale bar = 20 µm.

The differentiated Mstn^−/−^ clones exhibited marked morphological alterations, including myoblast fusion and myotube size. Therefore, phalloidin actin filaments and myosin heavy chain staining were employed to identify and quantify the thickness of the myotubes in both experimental groups. The initial observation demonstrated a marked increase in the size of the myotubes of the Mstn^−/−^ compared to Mstn^+/+^ differentiated cells after seven days (Figure 4a–d). The thickness of the myotubes was quantified in Mstn^−/−^ 18–19, Mstn^−/−^ 20–38 and Mstn^−/−^ 21–48 Mstn-edited clones by using morphometric analysis. The data indicated significant increases in the thickness of the myotubes of the Mstn^−/−^ clones (*p* < 0.001) compared to the Mstn^+/+^ induced cells (Figure 4e). To examine the capacity of muscle precursors to undergo differentiation in terms of forming multinuclear myotubes, the fusion index was assessed. The latter was calculated by dividing the number of nuclei per myotube by the total number of nuclei in the same microscopic field. The analysis demonstrated an increase in the fusion capacity of the differentiated Mstn^−/−^ cells, indicative of enhanced MD (*p* < 0.001), compared to the Mstn^+/+^ counterpart (Figure 4f).

## 4. Discussion

Genetic manipulation of myostatin has emerged as a promising approach for developing therapeutic strategies for muscle disorders, including cachexia, muscle wasting, muscle degeneration and muscular dystrophies. Previous reports have documented positive outcomes following either myostatin deletion or the blocking of its activity using various means, including antibodies [44,45], overexpression of the inactive propeptide domain [46,47,48], zinc finger nucleases in cattle [49] and CRISPR/Cas9 editing in guinea pigs [38]. Recently, CRISPR/Cas9 technology has emerged as a rapid, straightforward and precise method to generate gene deletion, requiring only a modification of the guide RNA to identify the target site [50]. The present study examines the impact of Mstn editing in muscle stem cells using the recent CRISPR/Cas9 on the physiological parameters and MD capacity. To this end, an immortalized, widely used mouse myoblast cell line, C2C12, was employed to assess the efficacy of myostatin CRISPR/Cas9 editing. A previous report has recommended the use of C2C12 as a stable primary cell line suitable for manipulating the Mstn signaling pathway [51].

The data analysis indicated the successful generation of multiple Mstn^−/−^-edited clones through the implementation of various molecular biology techniques. We were able to generate several Mstn knockout clones from a single nucleotide deletion, as shown in in the Mstn^−/−^ 18–20, up to a 243 nucleotide deletion in Mstn^−/−^ 17–9. Furthermore, the data analysis of the western blot experiment demonstrated the absence of Mstn precursor protein for the Mstn^−/−^ 18–19, 20–38 and 21–48-edited clones compared to the control. The combined evaluation of clone sequencing and protein blotting suggests an alteration of Mstn transcription, which results in either truncated or insufficient mRNAs for protein translation. Moreover, the presence of various nucleotides suggests genetic variability and has an impact on the physiological performance of the cells of these clones. In agreement with our data, it has been reported that the efficiency of Cas9-mediated editing is dependent on the detection of the sequence alteration and the subsequent analysis of the modified region [52]. A similar study has examined the effect of CRISPR/Cas9-mediated gene editing in chick embryos. The authors reported evidence of an effective gene deletion, including absence of the transcripts, deletion of the respective protein and loss of the predicted effect of the edited gene [53]. In the present results, a variability of nucleotide deletion between clones was observed, which resulted in either insufficient gene editing or the presence of Mstn precursor protein in the edited clones, as shown in Mstn^−/−^ 18–15 using western blot. It was found that the most common problems for CRISPR/Cas9 editing were the off-target activities of CRISPR/Cas9 and its improper matching with the gRNA [54]; these limitations can be addressed through careful selection and optimizing the condition for the guide RNA [52].

To evaluate the influence of Mstn editing on the physiological performance of the harvested clones, MTT and SRB assays were conducted. The data analysis revealed no alteration in total protein content indicative of cell number in the Mstn^−/−^ cells up to 48 h in the growth medium compared to Mstn^+/+^. These data suggest that cell proliferation could be temporarily diminished as a consequence of genetic manipulation. Furthermore, the measurement of cell protein content for up to 48 h may not have been sufficient to observe a detectable increase in cell number, as has been previously reported in a germline Mstn deletion study [55]. To eliminate the possibility of cytotoxicity, cell viability assessment was conducted at the same time points. The data analysis indicated enhanced metabolic activity for up to 48 h for both Mstn^+/+^ and Mstn^−/−^-edited clones, which suggests that no apoptotic activity was involved as a consequence of gene editing. In a similar study investigating the impact of Mstn deletion using CRISPR/Cas9 in C2C12 on the ischemia reperfusion injury condition in vitro, the authors demonstrated that Mstn deletion protects the cells from oxidative stress through modulating the activity of p38, thus reducing the intrinsic apoptotic activity [56].

MyoD is a specific muscle precursor marker and an indicator of the myogenic commitment of myoblasts [57]. Meanwhile, committed myoblasts express MyoD to initiate MD, the expression of myogenin represents the onset of differentiation, and thus, both markers can provide information about the capacity of cell differentiation [58]. The cells of the Mstn^−/−^ clones exhibited diminished MyoD expression in comparison to the Mstn^+/+^ cells in the growth medium. In contrast, all differentiated Mstn^−/−^ clones exhibited elevated MyoD expression following seven days in MD medium. This observation points out that Mstn editing promotes myoblasts commitment to the myogenic lineage. These findings align with the observed upregulation of the differentiation marker myogenin expression. The data indicate an enhanced capacity of MD at the molecular level in Mstn-edited clones. Conversely, it is well established that Mstn regulates muscle growth by downregulating MyoD and reducing the number of cells due to its effect on cell division [59,60]. The inhibitory effect of Mstn on cell number has been previously elucidated: a study demonstrated that Mstn increased the expression of the cell cycle inhibitor p21 and decreased the activity of the Cdk2 protein, thereby modulating the cell cycle progression of muscle myoblasts [17]. In the same line with our data, a recent finding demonstrated that Mstn inhibition using quercetin, a plant extract, is capable of upregulating myogenic marker expression in differentiated C2C12 cells, including MyoD and myogenin [61]. The present data suggest that the absence of the Mstn protein promoted MyoD and Myogenic expression which resulted in increased myoblast commitment and differentiation potential. The results revealed that the MyH1 and MyH7 expressions were upregulated at late differentiation in the Mstn^−/−^-edited clones, indicating the expression of both fast and slow muscle protein isoforms, respectively, compared to Mstn^+/+^ cells. It is well established that the embryonic Mstn deletion results in an increase in the percentage of fast muscle fiber population in mice, as previously reported by our group [55,62]. In the same line, postnatal Mstn blocking using a systemic administration of the inactive propeptide domain has been shown to increase the size of fast glycolytic muscle fibers [46]. In the current study, it was difficult to determine whether Mstn editing alters myosin heavy chain protein expression for Mstn^−/−^-edited clones, since both isoforms are expressed in vitro compared to a well-organized heterogeneous population of muscle fibers observed in the adult Mstn knockout mouse.

The analysis of the selected clones revealed increases in the size of the myotubes in conjunction with upregulated mTOR and MyH expression, indicating promoted myogenic differentiation compared to the control cells of Mstn^+/+^. The augmented size of the myotubes was accompanied by an increase in the nuclear fusion rate in the Mstn^−/−^-edited cells, which points out an enhanced myogenesis. In the same line, we have detected an interaction between Mstn and mTOR expression in terms of a 2-fold upregulation of mTOR expression in differentiated cells of Mstn^−/−^-edited clones. Consistent with the current literature, these data confirm the enhanced protein synthesis through mTOR signaling in the absence of Mstn, which results in increased muscle mass similar to that observed in double-muscling in cattle and mice [56,63,64,65]. On the other hand, it has been reported that the electrotransfer of Mstn protein in rats resulted in a reduction in muscle mass due to the inhibitory effect of Mstn on the Akt/mTOR signaling in skeletal muscle. Similarly, a study showed that an activated insulin-like growth factor-1 (IGF-1) pathway was detected in the muscle of Mstn knockout mice through Akt signaling, suggesting that the absence of Mstn improves muscle protein synthesis, whereas the in vitro overexpression of Mstn by adenovirus inhibited the IGF-1/Akt pathway and reduced myotube size [66]. In the same context, the in vitro differentiation of MSCs of Mstn knockout mice demonstrated increased myotube size, which suggests an increase in protein synthesis by targeting Akt/mTOR pathway [67]. Moreover, a study concluded that the activity of the Akt/mTOR pathway, including its downstream p70S6K and PHAS-1/4E-BP1 signaling cascade, plays an essential role in protecting the muscle fiber against muscle atrophy [68]. The increases in muscle mass following Mstn deletion could alter the physiological performance and the metabolic activity of the muscle. A study reported that although an increase in muscle mass was observed in the Mstn knockout mice, the force production was compromised, and the mitochondria were depleted [69]. In agreement with these results, a study found that Mstn knockout through a pronuclear microinjection modulates mitochondrial performance through inhibiting the phosphorylated adenosine monophosphate-activated protein kinase (pAMPK)/silent information regulator-1 (SIRT1)/peroxisome proliferator-activated receptor γ coactivator-1α (PGC1-α) pathway. The authors suggest that Mstn deletion regulates energy homeostasis [70]. Furthermore, using CRISPR/Cas9 Mstn editing inhibited the ATP synthesis by OXPHOS, indicating an alteration in the mitochondrial oxidative phosphorylation [71].

The data analysis revealed that ActRIIb was upregulated following myogenic differentiation in all Mstn^−/−^-edited clones, which may be attributed to the absence of the inhibitory effect of Mstn on the muscle cell when Mstn binds to its own receptor. The data suggest that the expression of ActRIIb was elevated to compensate the lack of Mstn activity, as well as to maintain a balanced signaling pathway. Furthermore, other TGF-β ligands, including activin and GDF-11, may contribute to the compensatory upregulation of the receptor expression. A study demonstrated that both Mstn and activin are capable of binding to both ActRIIa and ActRIIb; the blockade of one of these receptors through the use of specific antibody resulted in an incomplete loss of function and reduction in muscle mass. The author suggests that a double-blockade for both receptors may be a more effective approach to achieve the optimal response [72]. Similarly, the use of a soluble form of ActRIIb has been demonstrated to inhibit Mstn activity and resulted in a 60% increase in muscle mass; however, less effect was observed in the Mstn knockout mice, which suggests the involvement of other molecules in the regulation of muscle growth [73]. Moreover, a study reported that the blockade of ActRIIb was able to improve the phenotypic characteristics in the mouse model of DMD [74], as well as the mouse model of amyotrophic lateral sclerosis [75]. In contrast to our results, a study employing quercetin to eliminate Mstn in MSCs observed that quercetin-based Mstn inhibition resulted in an enhanced myogenic differentiation accompanied by a reduction in ActRIIb activity [61].

The interaction of decorin with the active myostatin ligand within the extracellular matrix may potentially inhibit its activity on muscle cells [76]. The present study revealed the downregulation of decorin expression in all differentiated Mstn^−/−^-edited clones. The data suggest that decorin expression was downregulated, as decorin typically exerts an inhibitory effect on the active domain of Mstn within the extracellular matrix. Therefore, in the absence of Mstn, the inhibitory effect of decorin was no longer provided. In the same line, a study demonstrated that decorin overexpression diminished myostatin activity, resulting in enhanced myoblast proliferation and differentiation in duck myoblasts. Additionally, the expression of decorin was downregulated toward the differentiation, similar to the data presented here [77]. Similarly, a study has indicated that the inhibitory effect of decorin on Mstn expression has a valuable impact on enhancing muscle differentiation and muscle regeneration by blocking Mstn activity [78]. Furthermore, another study has identified a regulatory mechanism between Mstn, TGF-β1 and decorin, which may modulate the fibrotic activity of Mstn in both muscle and fibroblast cells [79].

The immunocytochemistry analysis demonstrated no significant alteration in the number of MyoD-positive cells between the two genotypes. The number of myogenin-positive cells was found to be increased in Mstn^+/+^ under differentiation conditions. In contrast to the myogenin expression results, the number of myogenin-positive cells remained stable after seven days in the differentiation medium for Mstn^−/−^ cells, as determined using immunocytochemistry. The most plausible interpretation is that the evidence of myogenin expression either requires a long time to be detected at the protein level, or that Mstn deletion has a negative influence on the transition from MyoD to myogenin, which interferes with myogenin protein synthesis. In agreement with the second hypothesis, a study found that cells of the Mstn^+/+^ exhibited a more rapid withdrawal from the cell cycle and subsequent differentiation via upregulating the cell cycle inhibitor p21. In contrast, myoblasts from the Mstn^−/−^ group showed a delayed withdrawal from the cell cycle, a persistent expression of MyoD and an interruption in the expression of myogenin, which ultimately resulted in impaired myogenic differentiation [17].

## 5. Conclusions

The present report validates the possibility of Mstn editing via CRISPR/Cas9 technology, as well as the impact of Mstn deletion on the MD capacity of muscle myoblasts cell line. The objective was to target five loci of Mstn using guide RNA oligonucleotides through applying molecular tools, including cloning, transformation, transfection and the selection of specific clones. The latter sequencing demonstrates a successful editing of Mstn, as evidenced by the loss of nucleotides of variable length and the absence of Mstn precursor protein synthesis. The viability of the Mstn-edited clones remained stable, with no evidence of cytotoxicity. Mstn knockout muscle precursors exhibited enhanced myogenic commitments, increased protein synthesis and elevated myosin heavy chain expression. The Mstn knockout clones revealed an enhanced differentiation potential, as evidenced by augmented myoblasts fusion and an increased size of the myotubes. Collectively, the data indicate that Mstn editing via CRISPR/Cas9 could be a promising therapeutic strategy to enhance the regenerative capacity of muscle stem cells before in vivo application. Furthermore, targeting Mstn represents a promising avenue for developing effective treatments for muscular diseases, as well as for augmenting muscle mass in the context of wasting disorders.

## Figures and Tables

**Table 1:** Primers sequences used for the guide RNA targeting myostatin loci.

Myostatin Target Sequences	Vector	Primers
GCCAAGAGCGCCTCCACTCC GGG	pMH17	Forward: CACCGCCAAGAGCGCCTCCACTCC
		Reverse: AAACGGAGTGGAGGCGCTCTTGGC
GCGATCAGTACGACGTCCAGA GGG	pMH18	Forward: CACCGCGATCAGTACGACGTCCAGA
		Reverse: AAACTCTGGACGTCGTACTGATCGC
GTGACGATTATCACGCTACCA CGG	pMH19	Forward: CACCGTGACGATTATCACGCTACCA
		Reverse: AAACTGGTAGCGTGATAATCGTCAC
GAGGTGACAGACACACCCAAG AGG	pMH20	Forward: CACCGAGGTGACAGACACACCCAAG
		Reverse: AAACCTTGGGTGTGTCTGTCACCTC
GAAAGACGGTACAAGGTATAC TGG	pMH21	Forward: CACCGAAAGACGGTACAAGGTATAC
		Reverse: AAACGTATACCTTGTACCGTCTTTC

**Table 2:** Primers sequences used for PCR analysis.

Primer	Forward	Reverse
Myogenin	GAAGAAAAGGGACTGGGGAC	TCTTGAGCCTGCGCTTCTCC
MyoD	GTGAATGAGGCCTTCGAGAC	GAGCCTGCAGACCTTCGATG
Myostatin	ATGAGGGCAGTGAGAGAGAAG	CGCAGCTTACTGAGGATTTG
ActRIIB	AACCCCCAGGTGTACTTCTG	TGGCTCGTACGTGACTTCTG
Decorin	CCCGACACAACCTTGCTAG	CCTCTGGACTGATTTTGCTG
mTOR	CGGCACACATTTGAAGAAGC	TCCATGCTGCTGATACGAAC
MYH1	CTACAACATCGCTGGCTGG	GCCACCAGACTCTGCTTCC
MYH2	TGGCTGGCTGGACAAGAAC	TCCACCACTACTTGCCTCTG
MYH7	ACTATGCTGGAGCTGATGCC	CTCTGTGCAGAGCAGACAC
18S	ATGCGGCGGCGTTATTCC	GCTATCAATCTGTCAATCCTGTCC

**Table 3:** Sequencing of the myostatin-edited clones.

Clone	Nr. of Deleted Nucleoides
Mstn^−/−^ 17–9	243
Mstn^−/−^ 17–7	51
Mstn^−/−^ 21–48	38
Mstn^−/−^ 18–19	23
Mstn^−/−^ 20–38	5
Mstn^−/−^ 19–21	3
Mstn^−/−^ 18–20	1

## Data Availability

The data presented in this study are available upon reasonable request from the corresponding author. The data are not publicly available due to privacy reasons.

## Supplementary Materials

The following supporting information can be downloaded at www.mdpi.com/article/10.3390/jdb13010005/s1: Figure S1: Schematic illustration of the experimental procedures of CRISPR/Cas9 editing. Figure S2: Sequencing of single-cell myostatin-edited clones using CRISPR/Cas9. Figure S3: Western blot of myostatin precursor protein.

## Abbreviations

MSCs	Skeletal muscle-derived stem cells
PF	Proliferation condition
DF	Differentiation condition
MyoD	Myogenic determinant factor 1
MRF4	Myogenic regulatory factor 4
Pax7	Paired-box transcription factor 7
SRB	Sulforhodamine B
MTT	(3-(4,5-dimethylthiazol-2-yl)-2,5-diphenyltetrazolium bromide) cell viability assay
ActRIIb	Activin receptor type 2b
FGFs	Fibroblast growth factors
TGF-β	Transforming growth factor-beta
HGF	Hepatocyte growth factor
TNF-α	Tumor necrosis growth factor-alpha
IL-6	Interleukin-6
MyF5	Myogenic regulatory factor 5
MyH1	Myosin heavy chain 1
MyH2	Myosin heavy chain 2
MyH7	Myosin heavy chain 7
GDF-8	Growth and differentiation factor-8
GDF-11	Growth and differentiation factor-11
pAMPK	phosphorylated adenosine monophosphate-activated protein kinase
SIRT1	silent information regulator-1
PGC1α	peroxisome proliferator-activated receptor γ coactivator 1α
IGF-I	Insulin growth factor-I
mTOR	Mammalian target of Rapamycin
