# Supplementary material for: CRISPR/Cas9-Targeted Myostatin Deletion Improves the Myogenic Differentiation Parameters for Muscle-Derived Stem Cells in Mice
Source: J Dev Biol. 2025 Feb 11;13(1):5. doi: 10.3390/jdb13010005 (PMC11843916; doi:10.3390/jdb13010005)
Supplement: Supplementary file 1 [file jdb-13-00005-s001.zip › jdb-3375302-supplementary.pdf]

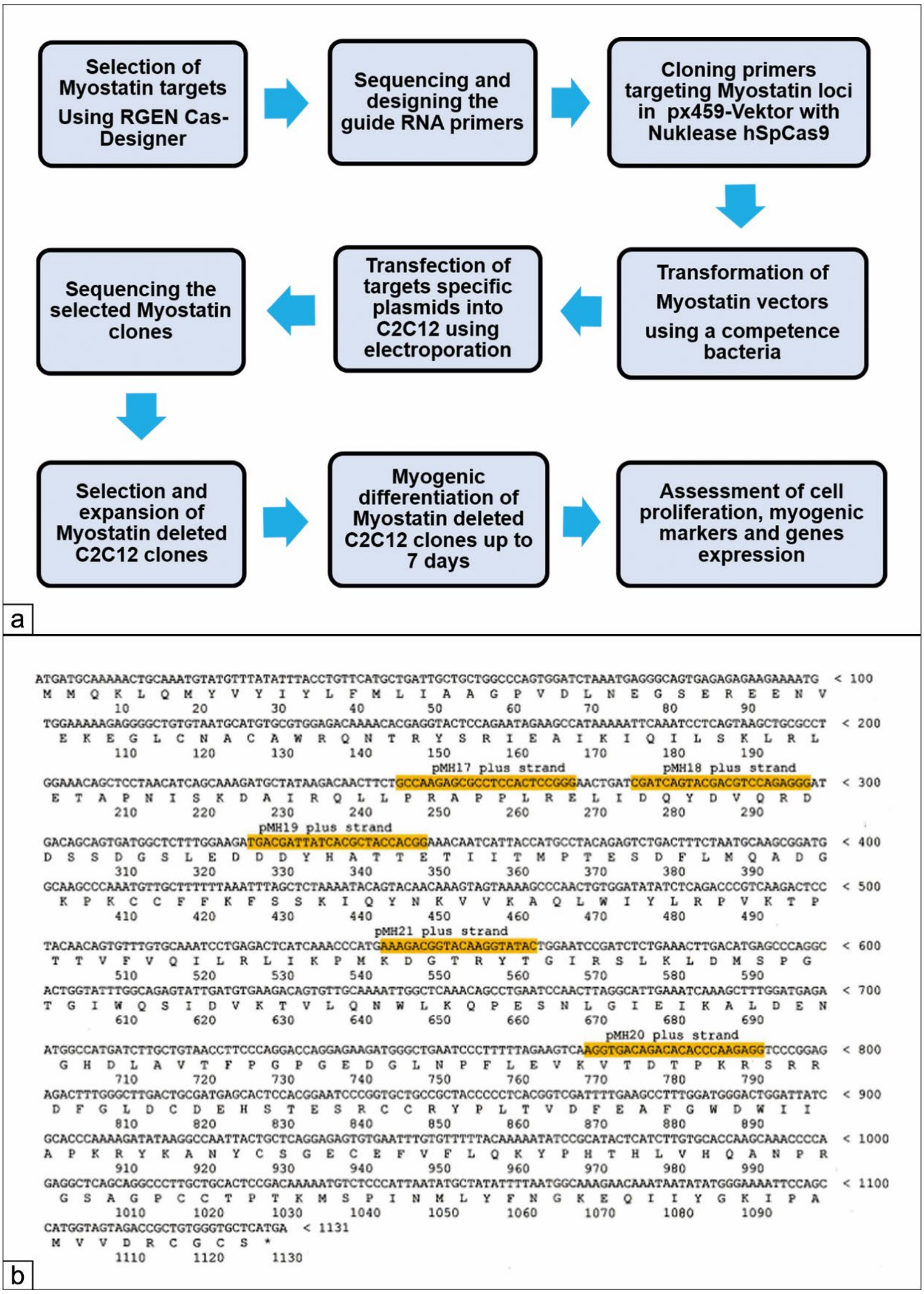

Figure S1. (**a**) Schematic illustration of the experimental procedures, including Selection of the Myostatin target loci, Designing the guide RNA oligonucleotide, the cloning of the selected DNA targets into p459 vector in conjunction with CRISPR/Cas9 endonuclease, transformation and transfection, single clone selection and expansion and myogenic differentiation of the Myostatin edited clones in comparison to C2C12 control. (**b**) An overview of five targeted sequences in the Myostatin based on NCBI (Reference Sequence: NM_010834.3 and serial cloner software.

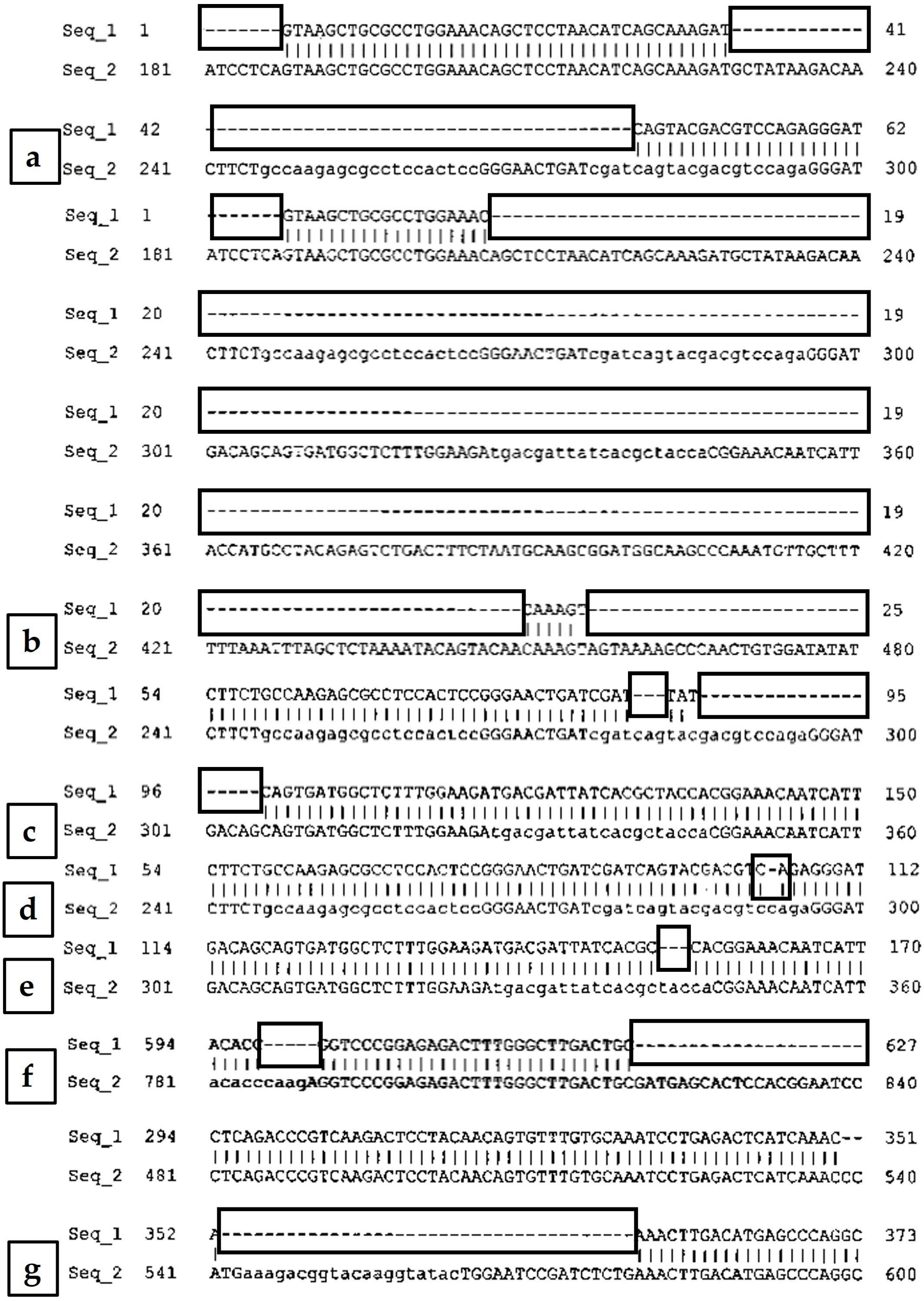

Figure S2. Sequencing of single-cell Myostatin-edited clones using CRISPR/Cas9. Analyzing of the sequences demonstrated marked nucleotides deletion (dark labelled squares) in (**a**) Mstn$^{-/-}$ 17-7, (**b**) Mstn$^{-/-}$ 17-9 (**c**) Mstn$^{-/-}$ 18-19, (**d**) Mstn$^{-/-}$ 18-20, (**e**) Mstn$^{-/-}$ 19-21, (**f**) Mstn$^{-/-}$ 20-38, and, Mstn$^{-/-}$ 21-48.

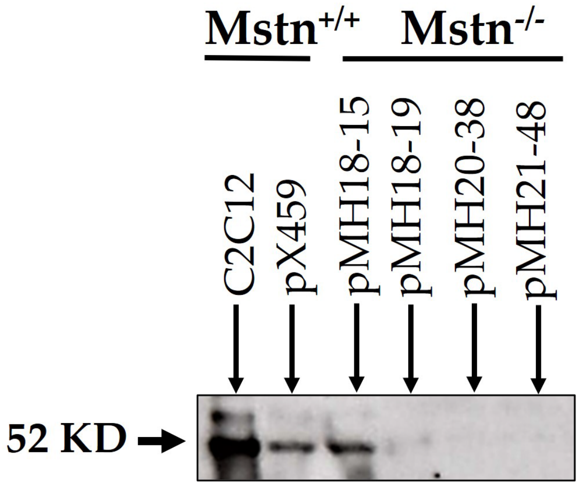

Figure S3. Western blot of myostatin precursor protein at 52 KD for C2C12 (Myostatin control, Mstn+/+), pX459 (Negative control vector) and CRISPR/Cas9 myostatin-edited clones (Mstn-/-) including pMH18-15, pMH18-19, pMH20-38, and pMH21-48. 2 × 104 C2C12 of Mstn+/+ and Mstn-/- edited clones. Cells were lysed after seven days in myogenic differentiation condition in lysis buffer comprising of of 1 M urea, 2% sodium dodecyl sulphate (SDS), 10% glycerin, 0.01% bromophenol blue and 6.25 mM Tris–HCl for 2 min. Protein samples for all experimental groups were loaded into 7.5 % SDS-polyacrylamide gel electrophoresis, then were transferred on nitrocellulose membranes (Pall Bio Trace) at 350 mA for 90 min in a blotting chamber (VWR, Darmstadt, Germany). The membrane was blocked with 5 % skimmed milk powder supplemented with 0.1% Tween in PBS (PBST) at 4 °C. The membrane was washed twice in PBST for 5 min, then were incubated with GDF-8/11 (H-9) mouse monoclonal anti-myostatin primary antibody diluted (1: 1000) in PBST (Santa Cruz Biotechnology Inc, Heidelberg, Germany) for 1 h at room temperature. The membrane was washed three times in PBST for 5 min, then were incubated with goat-anti mouse IgG (Dianova) horseradish-peroxidase-conjugated secondary antibody (1:5000) for 45 min. After five washing in PBST, the protein bands were visualized using ECL Select Western Blotting Detection Reagent (GE Healthcare, RPN2235,) with Amersham Hyperfilm (#28906836).
